# Detecting Change in Needs-Supplies Fit Through Reliable Change Methodology

**DOI:** 10.1177/00332941231212845

**Published:** 2023-11-03

**Authors:** Kleinjan Redelinghuys

**Affiliations:** Department of Industrial Psychology and People Management, 61799 University of Johannesburg, South Africa

**Keywords:** needs-supplies fit, person-environment fit, reliable change methodology, reliable change index

## Abstract

Studying change is a critical part of psychology and science in general. Studies often treat fit as static and use between-person designs to assess change. Accordingly, potentially insightful within-person information is frequently overlooked. The current study aimed to establish the utility of reliable change methodology within the domain of organizational psychology, using needs-supplies fit as a guiding framework. When employee needs can be tracked with a fair degree of clarity, organizations can devise better strategies to routinely address discrepancies between desired employee needs and organizational offerings. This longitudinal study used secondary data from 258 secondary school teachers. The Needs-Supplies Fit Scale was administered. The study’s hypothesis was assessed through reliable change methodology. When considering all the participants that experienced at least some change across time intervals (*n* = 148), 23.65% (*n* = 35) of this change was meaningful. This declined to 17.33% meaningful change when factoring in the entire sample, including those who did not experience any change. When organizations are aware of the ever-evolving needs of employees, quicker action can be taken to avoid impending person-environment misfit. This study contributes to existing within-person studies that showcased the malleability of needs-supplies fit and emphasize the value of placing a more prominent focus on the individual.

## Introduction

Studying change is a critical part of psychology and science in general (Blampied, 2022; Estrada et al., 2019). Unfortunately, studies often list cross-sectional research designs as a limitation as it inhibits us from tracking how constructs change over time. Consequently, in many instances, constructs like person-environment fit are treated as static, when in fact they could be changeable to a certain extent (Vleugels et al., 2019). Another possible limitation, although from a longitudinal perspective, is to draw conclusions from between-subject statistics and overlook potentially useful within-subject statistics (Vleugels et al., 2022), which may provide an obstructed view of how constructs change. One way to determine if constructs show meaningful change at the individual level is to employ reliable change methodology. The purpose of this method is to determine whether an individual’s change score from one measurement point to another is of such a magnitude that it goes beyond change that is solely attributed to measurement error (Jacobson & Truax, 1991). Although reliable change methodology is commonly used in various branches of psychology, like clinical psychology and neuropsychology (e.g., Karr et al., 2021; Van Patten et al., 2021), its application is less evident in organizational psychology. Therefore, the current study aimed to establish the utility of reliable change methodology within the domain of organizational psychology, using needs-supplies fit as a guiding framework.

### Possible utility of reliable change methodology in organizational psychology

To understand the value of assessing reliable change in organizational psychology, it may be useful to briefly cover its origins and explore how it is utilized in other contexts. Within the confines of clinical psychology, Jacobson and colleagues (Jacobson et al., 1984; Jacobson & Truax, 1991) pioneered the concept of reliable change and its underlying methodology. They viewed it as the initial stage in evaluating psychotherapy’s efficacy, a prerequisite in establishing if therapy had brought about clinically significant change (Wise, 2004). Hence, when individuals met certain criteria during the course of therapy, they could be placed in different classification categories, such as *recovered* (meeting criteria for clinically significant and reliable change), *improved* (meeting criteria for positive reliable change), or *deteriorated* (meeting criteria for negative reliable change) (Jacobson & Truax, 1991; Wise, 2004). Individuals who met none of the preceding criteria were classified as *unchanged/indeterminate* (Jacobson & Truax, 1991; Wise, 2004). Reliable change methodology was later adopted by neuropsychologists, predominantly to ascertain if individuals who suffered neurological setbacks experienced changes over time that were large enough to signify improvement or deterioration. Thus, in neuropsychology, it may aid clinicians in (a) monitoring how individuals recuperate from injuries, (b) tracking neurodegenerative diseases, and (c) assessing the efficacy of neuropsychological interventions (Karr et al., 2021).

As seen in the previous paragraph, reliable change methodology could be valuable in answering a variety of research questions across multiple disciplines. In the context of organizational psychology, the constructs being scrutinized, the research objectives, and the study design would largely dictate how reliable change methodology could be of value. For example, in some cases, organizational psychologists might follow a similar approach to clinical psychologists to determine if an individual recovered, improved, or deteriorated following interventions to treat burnout, for example. In other instances, organizational psychologists might follow an approach that is more aligned with neuropsychologists to establish if individuals improved or deteriorated in the change they experienced on a particular construct, without an intervention component. Accordingly, this may aid organizations and organizational psychologists in determining if it is necessary to intervene when individuals experience a decline in their motivation, work engagement, job satisfaction, or any other relevant employee behaviors, attitudes, perceptions, or intentions that might adversely affect the individual or the organization.

The latter approach to reliable change served as a basis for the current study, although the functional deterioration from a neuropsychological perspective was of no concern here. In the context of needs-supplies fit, reliable change methodology could offer a better understanding of the malleability of needs-supplies fit. When employee needs can be tracked with a fair degree of clarity, organizations can devise better strategies to routinely address discrepancies between desired employee needs and organizational offerings. By putting these tactics into practice, employees and organizations should reap the benefits that often accompany person-environment fit (Edwards & Shipp, 2007), such as job satisfaction, life satisfaction, organizational citizenship behavior, work engagement, and work-related wellness (Gander et al., 2020; Morrow & Brough, 2019; Stone et al., 2019). Furthermore, this could also ensure that organizations do not devote unnecessary time and resources to address change that is not large enough to cause concern.

### Needs-supplies fit as a type of person-environment fit

The utility of person-environment fit in the domain of organizational psychology is well documented (Guan et al., 2021). Person-environment fit generally reflects a sense of harmony between employees and their work environment based on certain characteristics (Kristof-Brown & Guay, 2011). Consequently, employees may experience fit with different segments of their organizational environment, which may include the organization itself, the people they interact with at work (e.g., supervisor, their workgroup), and their job (Jansen & Kristof-Brown, 2006). Needs-supplies fit, often seen as part of the person-job fit component within person-environment fit theory, captures employee perceptions of their needs being met by organizational offerings (Cable & DeRue, 2002). Needs-supplies fit is often combined with demands-abilities fit to form person-job fit (e.g., Brkich et al., 2002). From a theoretical and statistical viewpoint, several studies have however shown that needs-supplies fit and demands-abilities fit should be differentiated (e.g., Cable & DeRue, 2002), as the manner in which they influence other variables may differ (e.g., Morrow & Brough, 2019). Hence, the current study isolated needs-supplies fit from other types of person-environment fit.

When it comes to person-environment fit research, Gabriel et al. (2014) observed that “Almost all of the research on fit perceptions has been conducted at the between-person level of analysis, which implicitly ignores the possibility of substantive within-person changes” (*p*. 390). This statement was largely supported by findings of a systematic review study that focused on temporal aspects related to person-environment fit research (Vleugels et al., 2022). From the studies included in their review, Vleugels et al. (2022) found that most longitudinal studies had a between-person design (73.91%), as opposed to a within-person study design (10.87%), or other study designs (e.g., qualitative). Those who employed longitudinal within-person study designs mainly used cluster analysis, hierarchical linear modeling, multilevel path analyses, nonparametric pairwise difference tests, and polynomial regression to analyze their data (Swider et al., 2015; Tepper et al., 2018; Vleugels et al., 2018, 2019; Vogel et al., 2020). The purpose of the preceding studies was to assess the dynamic nature of aspects of person-environment fit in relation to other constructs.

In another study, Kim et al. (2020) used mixed-effects growth models and hierarchical multiple regression to assess the dynamic relationships between demands-abilities fit, needs-supplies fit, person-job fit, and employee outcomes (job satisfaction and organizational commitment). They utilized a three-wave design, with six-month time intervals between Time 1 and 2, as well as between Time 2 and 3. Findings from their study showed that when job supplies increased, perceived needs-supplies fit increased. From the seven types of needs (autonomy, pay, prestige, span of control, travel, vacation time, variety) assessed in the study, only an increase in prestige resulted in a significant increase in needs-supplies fit over time. Furthermore, Kim et al. (2020) found that an increase in employee needs led to a decrease in needs-supplies fit. Hence, excess supplies had a more desirable effect on needs-supplies fit as opposed to needs deficiency over time.

By applying reliable change methodology, the current study mainly deviates from the aforementioned studies as it primarily focuses on detecting within-person change on the concept of needs-supplies fit itself, and not how needs-supplies fit change in relation to other constructs over time. This will provide an indication of whether perceptions of fit change in a positive or negative direction. Ascertaining whether employees’ perceptions of fit change positively or negatively may offer an indication of whether they are potentially heading towards experiencing better fit or misfit.

Another deviation from prior studies (except for Kim et al., 2020) pertains to the time lag of the current study. Previous studies have mainly used daily or weekly time lags (e.g., Vleugels et al., 2018, 2019) to assess perceived fit. Findings from these studies showed within-person change that varied from 21% to 23% for needs-supplies fit, 21%–24% for person-organization fit, and from 28% to 40% for demands-abilities fit. As needs-supplies fit showed the lowest percentage of within-person change as per Cable and DeRue’s (2002) conceptualization and measurement in previous studies (Vleugels et al., 2018, 2019), it was decided that it would be best suited to use it as a guiding framework. This rests on the notion that, based on past experience, if the utility of reliable change can be shown for needs-supplies fit, its utility is likely to extend to other types of fit (e.g., person-organization fit and demands-abilities fit) too. Hence, numerous studies have shown that needs-supplies fit and other person-environment fit dimensions may change at short notice. To complement existing findings, this study set forth to investigate the evolvement of needs-supplies fit by examining within-person change over a six-month time interval; leading to the following hypothesis:

Hypothesis 1 The application of reliable change methodology will show that some employees display reliable change over time in needs-supplies fit while others do not, although they have non-zero change scores.

## Method

### Participants

Originally, 258 secondary school teachers from two South African school districts participated in the study, although only 202 completed the surveys at both time points. This resulted in a dropout rate of 21.71%. The sample was largely white (*n* = 156, 77.2%) female (*n* = 150, 74.3%) employees who were permanently employed (*n* = 170, 84.2%). Upwards of 50% of employees were employed for longer than ten years (*n* = 105, 52%) and were older than 35 (*n* = 103, 51%).

### Measuring instrument

The 3-item needs-supplies fit scale (Cable & DeRue, 2002) was used to measure needs-supplies fit. Its response options range from 1 (*strongly disagree*) to 7 (*strongly agree*). A sample item reads as follows: ‘The job that I currently hold gives me just about everything that I want from a job’. Various studies have found the subscale to be reliable in cross-sectional (e.g., Carstens et al., 2021; Van Zyl et al., 2022) and longitudinal (e.g., Vleugels et al., 2018) settings. Consequently, the following reliability coefficients were found: α = .91 (Carstens et al., 2021), ω and α = .90 (Van Zyl et al., 2022), and average α = .95 (Vleugels et al., 2018).

### Procedure

The researcher collected the primary data, captured it in Excel, anonymized the data, and stored it on a password-protected computer and secure cloud service. The initial paper-based surveys were destroyed after 5 years. The current study used secondary data to examine a hypothesis that differed from any of the hypotheses that were explored by other studies using the primary dataset (see Redelinghuys et al., 2019a, 2019b; Redelinghuys & Rothmann, 2020). More information about the original research procedure is reported by Redelinghuys (2016).

### Ethical considerations

Redelinghuys (2016) obtained ethical clearance to collect primary data (Ref: NWU-HS-2015-0193). To use the dataset for secondary purposes, an ethics application was submitted to the Department of Industrial Psychology and People Management’s Research Ethics Committee, whereby ethical approval (Ref: IPPM-2023-722) was given. During initial data collection, participants received an information leaflet that explained the study’s purpose and the requirements for participation. Those who were willing to partake in the study were asked to sign a consent form. The secondary dataset only included the responses from the participants who granted permission for their data to be used for future research purposes.

### Data analysis

R version 4.2.1 (R Core Team, 2022) and SPSS 28 (IBM Corp, 2021) were used to perform different types of statistical analyses. Within RStudio (RStudio Team, 2022), the following R packages were utilized: *MBESS* (Kelley, 2022), *psych* (Revelle, 2022), and *stats* (R Core Team, 2022). The analyses, packages, and criteria for statistical significance are discussed in greater detail in the results section. This serves the purpose of making the results easier to interpret.

## Results

### Descriptive statistics and reliability

Cronbach’s alpha coefficients for the needs-supplies fit subscale were calculated with the *ci.reliability* function in the *MBESS* (Kelley, 2022) package. Other descriptive statistics were computed with the *describe* function from the *psych* (Revelle, 2022) package. Table 1 presents the reliability coefficients and descriptive statistics.

**Table 1.** Reliability Coefficients and Descriptive Statistics for Needs-Supplies Fit.

Construct	α	Mean	*SD*	Skew	Kurt
Needs-supplies fit (time 1)	.91	5.02	1.38	−.86	.27
Needs-supplies fit (time 2)	.96	4.91	1.43	−.59	−.58

*Note.* α = Cronbach’s alpha coefficient; *SD* = standard deviation; Skew. = skewness; Kurt. = kurtosis.

Examination of Table 1 shows that the reliability coefficients exceeded Nunnally’s (1978) suggestion of .80 for research purposes. Skewness and kurtosis values fell within acceptable ranges (−2 to 2) of univariate normality (Koh, 2014).

### Reliable change methodology

To assess whether there was a significant mean difference between needs-supplies fit during Time 1 and 2, a paired sample *t* test was conducted using the *t.test* function from the *stats* package (R Core Team, 2022). The results revealed no significant mean difference: *t* = 1.3612, *df* = 201, *p* = .175, x̄ difference = .09 [95% CI = −.05, .27]. Although the preceding result was not statistically significant, it did not rule out potential significant individual differences. Therefore, to analyze individual differences, each individual’s composite score obtained during Time 2 was deducted from the score they obtained during Time 1. Of these 202 observations, 54 observations remained the same, whereas 148 (73.27%) presented difference scores. Despite these differences, it was still unclear to which extent they were statistically meaningful. Consequently, reliable change methodology was used.

When applying reliable change methodology, it is useful to distinguish between reliable change and the reliable change index (RCI; Christensen & Mendoza, 1986; Jacobson et al., 1984; Jacobson & Truax, 1991). Although both yield similar conclusions (Blampied, 2022), they differ conceptually. Reliable change is calculated by dividing an individual’s change score (e.g., Time 1 raw score – Time 2 raw score) by the Standard Error of the Difference (*S*_Diff_; see p. 14 of Jacobson & Truax, 1991). “This converts the raw change score to standard deviation (specifically *S*_Diff_) units, just as a *z*-score is the difference between an individual’s score and the mean standardized by the *SD*” (Blampied, 2022, p. 5). Hence, reliable change is an individual attribute. In contrast, the RCI is an attribute of a measuring instrument. It offers a way to calculate an index score for a specific measuring instrument, indicating the absolute difference score that is required for change to be reliable at a specified probability level (Blampied, 2022). For example, at the *p* < .05 probability level, the RCI is calculated by multiplying 1.96 by the Standard Error of the Difference (see p. 6 of Blampied, 2022; formula 6).

From the preceding paragraph, the difference between reliable change and the RCI is relatively clear. However, the best method for calculating the Standard Error of the Difference is still debatable. This problem is frequently encountered when applying reliable change methodology, leading to varied RCIs, and often to different conclusions (Guhn et al., 2014). Therefore, to avoid confusion, the steps outlined by Iverson (2011, 2012) were followed in this study. Before making any calculations, the following values were required: the standard deviation for needs-supplies fit at both time points and the test-retest coefficient. The standard deviations were required to calculate the standard error of measurement for both time points. The *sd* function from the *stats* (R Core Team, 2022) package was used to calculate the standard deviation for Time 1 (1.43) and Time 2 (1.38). An intraclass correlation coefficient (ICC) was calculated in SPSS (IBM Corp, 2021) as a representation of the test-retest coefficient, using a two-way mixed model with an absolute agreement type. An intraclass correlation of .80 [95% CI = .73, .85] was established. When using Koo and Li’s (2016) recommendation, this falls within the good test-retest reliability range (.75–.90). Table 2 displays the step-by-step process followed in calculating the RCI as adapted from Iverson (2011, 2012).

**Table 2.** Sequential Steps in Calculating the RCI.

Steps	Formula	Calculation in R using input values from the current study
Step 1 – Calculate the standard error of measurement (denoted as SEM_1_) for needs-supplies fit (time 1)	Standard deviation (SD) of needs-supplies fit (time 1) multiplied by the square root of 1 minus the value of the test-retest coefficient	1.43*sqrt (1–.80) = .64
Step 2 – Calculate the standard error of measurement (denoted as SEM_2_) for needs-supplies fit (time 2)	Standard deviation (SD) of needs-supplies fit (time 2) multiplied by the square root of 1 minus the value of the test-retest coefficient	1.38*sqrt (1–.80) = .62
Step 3 – Calculate the standard error of the difference (denoted as SE_diff_)	Square root of the sum of the squared SEMs for time 1 and 2	sqrt (.64^2^ + .62^2^) = .89
Step 4 – Calculate the reliable change index (RCI)	Standard error of the difference (SE_diff_) multiplied by a z-score: 1.96 (95% confidence interval), 1.645 (90% confidence interval), 1.282 (80% confidence interval), and 1.04 (70% confidence interval)	.89 × 1.96 = 1.74 .89 × 1.645 = 1.46 .89 × 1.282 = 1.14 .89 × 1.04 = .93

Table 2 shows an RCI value of 1.74 (95% confidence interval) when applying strict criteria to assess reliable change. Similarly, more lenient scores of 1.46, 1.14, or .93 could be used as benchmarks to interpret meaningful change. Due to the non-clinical nature of the construct being studied and in line with other studies (Duff, 2012), it was decided that a 90% confidence interval would suffice. This meant that a change score of 1.46, in a positive or negative direction, was seen as statistically meaningful. Based on the preceding threshold, Table 3 reports the percentage of participants whose perceptions of needs-supplies fit meaningfully improved or declined.

**Table 3.** Percentage of Meaningful Change in Needs-Supplies Fit Scores.

Percentage improved (score >1.46)	Percentage declined (score < −1.46)
34.29% (*n* = 12)	65.71% (*n* = 23)

Table 3 indicates that a total of 35 participants experienced meaningful change according to the 90% confidence interval cut-off value, meaning that from Time 1 to 2, their scores on needs-supplies fit changed positively or negatively by a value that equaled or exceeded 1.46. Of these 35 participants, 12 experienced an improvement in needs-supplies fit, and 23 experienced a deterioration in the fit they experienced. When considering all the participants that experienced at least some change across time intervals (*n* = 148), 23.65% (*n* = 35) of this change was meaningful. This declined to 17.33% meaningful change when factoring in the entire sample, including those who did not experience any change. This offers support for the study hypothesis.

Although studies employing reliable change methodology often have to consider the impact of practice effects, for example, when assessing intelligence (Iverson, 2012), it is not relevant in the assessment of needs-supplies fit. This is due to the perception-based nature of needs-supplies fit, where questions are framed in such a way that one is incapable of answering correctly or incorrectly. Furthermore, due to the non-clinical nature of needs-supplies fit, establishing cut-off points for clinical significance was not relevant. Within organizational psychology, it may become relevant to assess clinical significance when assessing something like burnout, as studies have shown that the latter significantly associates with anxiety and depression (Koutsimani et al., 2019).

## Discussion

The current study aimed to establish the utility of reliable change methodology within the domain of organizational psychology, using needs-supplies fit as a guiding framework.

Regarding reliable change methodology, which formed the focal point of the study, results showed that roughly 17.33% of participants in the full sample experienced meaningful change. Although the methods employed in the current study are not equivalent to that of previous studies, Vleugels et al. (2018, 2019) found within-person change that varied from 21% to 23% for needs-supplies fit. Therefore, findings from this study and other studies acknowledge that needs-supplies fit is changeable to a certain extent, echoing Kim et al. (2020) sentiments that “Organizational life is not static, and characteristics of the work environment and employees change” (p. 291). Furthermore, results showed that of the participants who experienced change, 34.29% experienced improved scores, whereas 65.71% of participants’ scores declined. These percentages could not be compared to previous studies. Although one can only speculate about the reasons for a larger percentage of participants who declined, Kim et al. (2020) suggest that as employees’ careers progress, their needs are bound to change (e.g., expecting better compensation or increased autonomy) and sufficiently attending to these needs can be a timely process. This may among others be attributed to organizational policies, practices, or budgets that need to be changed or organizational resistance that needs to be subdued to meet employee needs (Kim et al., 2020). Hence, as long as this mismatch continues, one may expect decreased needs-supplies fit.

### Implications

Studies often treat fit as static and use between-person designs to assess change. Accordingly, potentially insightful within-person information is frequently overlooked. This study contributes to existing within-person studies that showcased the malleability of needs-supplies fit and emphasize the value of placing a more prominent focus on the individual. When organizations are aware of the ever-evolving needs of employees, quicker action can be taken to avoid impending person-environment misfit. This is especially important as an oversupply or undersupply in response to a specific need may under certain circumstances trigger undesirable employee responses (Van Vianen, 2018), for example, fatigue and reduced levels of engagement (Vogel et al., 2020). Although excess supplies are not always harmful and under certain conditions may address a variety of needs simultaneously (e.g., Kim et al., 2020), careful consideration is still required to harmonize employee needs and organizational offerings to achieve outcomes that are equally beneficial to employees and the organization. Additionally, through reliable change methodology, organizations can save valuable time and resources to only address real employee change as opposed to change, that is, solely ascribed to measurement error.

### Limitations and recommendations

Numerous study limitations surfaced. Firstly, needs-supplies fit was assessed in broad terms. This meant that although it could be ascertained that needs-supplies fit is malleable to a certain degree, it could not be ascertained if certain needs or supplies are more changeable than others. When measuring needs-supplies fit commensurably, some within-person studies (e.g., Tepper et al., 2018; Vogel et al., 2020) have shown that needs and supplies may vary somewhat. For example, when measuring the need for transformational leadership and the supply of transformational leadership received, Tepper et al. (2018) in their first study found the following within-person variance: need for transformational leadership (32.2%), supply of transformational leadership (51.3%). In another study, Vogel et al. (2020) found within-person variance of 37.6% for the need for daily meaningful work and 30.2% for the supply of daily meaningful work. Valuable insights can be obtained by assessing which combination of commensurate needs and supplies is more stable, and which is more changeable, using longitudinal designs with shorter and longer time intervals. In line with Kim et al.’s (2020) research, future studies could also further explore the effect of excess supplies and excess needs on needs-supplies fit over time based on a variety of commensurable needs and supplies.

Furthermore, results showed that the participants who remained part of the study experienced significantly higher scores on needs-supplies fit in comparison to those who dropped out. The possibility exists that this might have impacted estimates of change, although it would be hard to determine to what extent.
